# Amniotic stem cells as a source of regenerative medicine to treat female infertility

**DOI:** 10.1007/s13577-022-00795-1

**Published:** 2022-10-17

**Authors:** Aisha Naeem, Nikita Gupta, Usra Naeem, Mohamed A. Elrayess, Chris Albanese

**Affiliations:** 1 Department of Oncology, Lombardi Comprehensive Cancer Center, Georgetown University Medical Center, Washington DC, DC USA; 2 Health Research Governance Department, Ministry of Public Health, Doha, Qatar; 3 University of Lahore, Lahore, Pakistan; 4 Biomedical Research Center, Qatar University, Doha, Qatar; 5 College of Pharmacy, QU Health, Qatar University, P.O. Box 2713, Doha, Qatar; 6 Department of Radiology, Georgetown University Medical Center, Washington DC, DC USA; 7 Center for Translational Imaging, Georgetown University Medical Center, Washington DC, DC USA

**Keywords:** Infertility, Endometrium, Amniotic stem cells, Ovarian physiological aging, Ovarian failure

## Abstract

Impaired reproductive health is a worldwide problem that affects the psychological well-being of a society. Despite the technological developments to treat infertility, the global infertility rate is increasing significantly. Many infertility conditions are currently treated using various advanced clinical approaches such as intrauterine semination (IUI), in vitro fertilization (IVF), and intracytoplasmic injection (ICSI). Nonetheless, clinical management of some conditions such as dysfunctional endometrium, premature ovarian failure, and ovarian physiological aging still pose significant challenges. Stem cells based therapeutic strategies have a long-standing history to treat many infertility conditions, but ethical restrictions do not allow the broad-scale utilization of adult mesenchymal stromal/stem cells (MSCs). Easily accessible, placental derived or amniotic stem cells present an invaluable alternative source of non-immunogenic and non-tumorigenic stem cells that possess multilineage potential. Given these characteristics, placental or amniotic stem cells (ASCs) have been investigated for therapeutic purposes to address infertility in the last decade. This study aims to summarize the current standing and progress of human amniotic epithelial stem cells (hAECs), amniotic mesenchymal stem cells (hAMSCs), and amniotic fluid stem cells (hAFSCs) in the field of reproductive medicine. The therapeutic potential of these cells to restore or enhance normal ovarian function and pregnancy outcomes are highlighted in this study.

## Introduction

Over the past few daces, a substantial decline in fertility rate is observed around the world [1]. Both economic and reproductive health contributed significantly in poor fertility outcomes. Many pathological conditions could result in unsuccessful pregnancy outcomes such as premature ovarian failure/insufficiency (POF/POI), intrauterine adhesion (IUA), ovarian physiological aging (OPA), and disease-related infertility. Technologically advanced clinical approaches such as adhesiolysis, intrauterine semination (IUI), in-vitro fertilization (IVF), fertility preservation, and intracytoplasmic injection (ICSI) have helped manage fertility outcomes to a great extent, however, many issues remain to be addressed [2]

Despite the availability of cutting-edge therapies to restore or enhance infertility, some pathological conditions such as the dysfunctional uterus, persistent atrophic/thin endometrial lining, and loss of regeneration capacity of endometrial tissue lower the success rate of these treatments. For instance, successive embryo implantation failure due to a dysfunctional uterus or immune rejection remains the main reason for IVF treatment failure. Similarly, the inefficient process of endometrial tissue regeneration due to the loss of stem cells in the basalis layer of endometrium leads to pathological conditions such as intrauterine adhesion (IUA) or endometrial atrophy. Stem cell-based regenerative therapies hold the great capability of replenishing the functional deficit cell reservoir to address such pathological conditions [2].

Due to the differentiation ability into germ cells and oocyte-like cells, stem cells may adopt the following mechanisms to repair ovarian functions; i) heal injured reproductive tissues by replenishing healthy cells, ii) restore or increase the number of secondary and mature follicles, iii) improve microenvironment by secreting paracrine factors and ameliorate ovarian function, iv) immune regulation by secreting anti-inflammatory factors, and v) regulate the hormonal levels that maintain estrous reproductive cycles and stimulate ovulation such as E2 (Estradiol), AMH (Anti-Müllerian hormone), and FSH (Follicle-stimulating hormone). Despite the great success of stem cell therapeutics in reproductive disease management, the ethical concerns, heterological nature, low yield, and lower ex-vivo proliferation rate of adult stem cells limit their clinical translation [3]. Conversely, easily accessible placental-derived amniotic stem cells (ASCs) represent a viable therapeutic option due to their successful uses in other diseases and their differentiation ability toward cells of germline lineage [4–6]

**Fig. 1:**
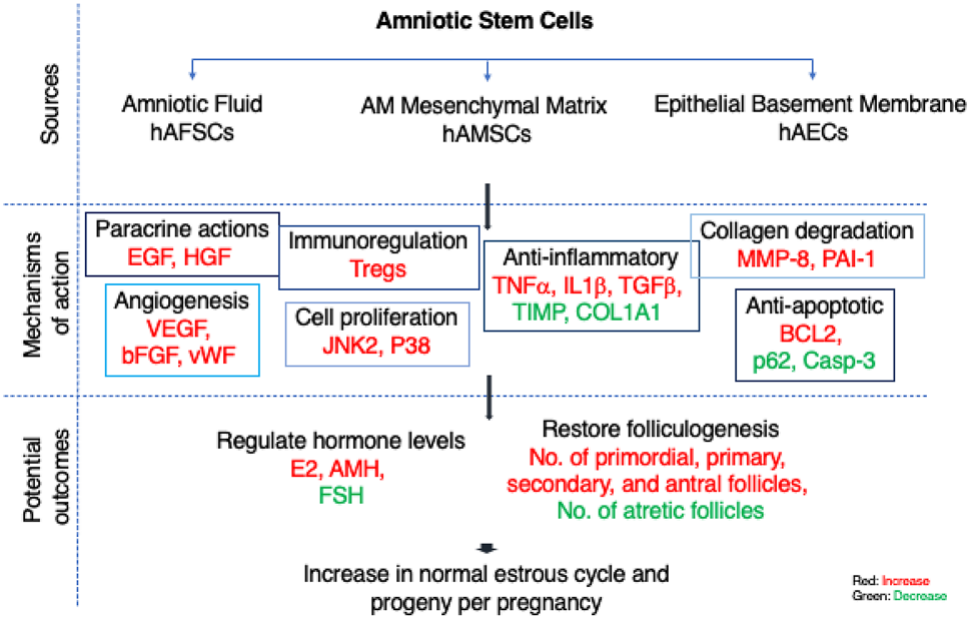
Schematic diagram illustrating the possible mechanisms for the restorative effects of amniotic stem cells. *AM* amniotic membrane; *AMH* anti-Müllerian hormone; *bFGF* basic fibroblast growth factor; *BCL*2 B-cell lymphoma 2; Casp-3 Caspase-3; *COL*1A1 collagen Type I Alpha 1 Chain; *EGF* epidermal growth factor; *E*2 Estradiol; *FSH* follicle-stimulating hormone; HGF hepatocyte growth factor; Tregs regulatory T cells; TIMP a tissue inhibitor of metalloproteinases; *TNFα* tumor necrosis factor alpha; *IL*1*β* interleukin 1 beta; *MMP*8 metalloproteinase-8; *PAI*-1 plasminogen activator inhibitor-1; TGF*β* Transforming growth factor beta; *vWF* Von Willebrand factor; *VEGF* vascular endothelial growth factor

Stem cells isolated from the umbilical cord have well-established therapeutic uses and are discussed in literature excessively [7]. Other emerging human ASCs that are currently being explored to treat infertility include amniotic epithelial stem cells (hAECs), amniotic mesenchymal stromal/stem cells (hAMSCs), and amniotic fluid stem cells (hAFSCs). However, no recent reports exist summarizing the progress of these stem cells in the field of reproductive disability. In this review, we revisited studies supporting the use of hAECs, hAMSCs, and hAFSCs to address various pathophysiological infertility conditions and briefly commented on the outcomes and limitations of the studies. Figure 1 presents the summarized view of potential mechanism of actions of ASCs to enhance fertility outcomes.

### Human amniotic mesenchymal stromal/stem cells (hAMSCs)

Mesenchymal stromal/stem cells (MSCs) derived from the avascular mesenchymal matrix of the human amniotic membrane (hAM) offer a beneficial option to replace adult MSCs [8–11]. Limited ex vivo proliferation, and complex sample retrieval limit the broad-scale utilization of adult MSCs [8]. The hAM is an easily accessible source of mesenchymal stem cells (hAMSCs) which overcomes these limitations. MSCs originating from hAM share many similarities with adult tissue-derived MSC and meet all the international standards for declaring them as “mesenchymal stem cells” [12]. The differentiation potential of hAMSCs has been discussed recently [8, 13]. Although the potential of adult MSCs in restoring ovarian function has been explored extensively [14–16], very limited data are available for AM-derived MSCs.

The studies that explored the role of hAMSCs to promote ovarian function in natural or premature ovarian aging (NOA or POA) or premature ovarian failure/inefficacy (POF/POI) mouse models are listed in Table 1. In these investigations, the hAMSCs demonstrated their potential to reinstate normal ovarian functions by improving the local microenvironment of the ovaries [17], maintaining endometrial regeneration through paracrine actions [13, 18–20], and/or regulating cytokines [21, 22] (Table 1, Fig. 1). Significant improvements in ovarian function were observed at the physiological and molecular levels following hAMSCs transplantations. The gain in ovarian function, thus fertility, was due to improved ovarian morphology, an increase in follicle count, a recovered estrous cycle, and improved levels of hormones (e.g., AMH) [17]. The paracrine factors such as EGF (epidermal growth factor) and HGF (hepatocyte growth factor), secreted by hAMSCs were shown to restore the hormonal level and follicle counts, however, the authors did not report subsequent fertility rate, gain in progeny, or improvement in estrous cycle regularity [21]. The noticeable improvements in total follicle count and hormonal levels were observed due to the occurrence of molecular events such as inhibition of cell apoptosis and induction of cell proliferation [17, 21]

**Table 1:** A list of in vivo studies demonstrated the use of amniotic stem cells to treat female infertility

Cell types	Studies	Infertility condition	Mouse model	Treatment groups	Reported markers	Post transplantation (PTP) outcomes	Limitations and unfavorable outcomes
Human amniotic mesenchymal stem cells	Fouad 2016	POF/POI	Mature white fertile albino rats CTX treated	hAMSC AD-MSCs	PTP: 30d ↑OCT4, CD29, Stra8	PTP: 15, 30d ↑E2 levels, no. of mature follicles and corpora lutea with oocytes ↓FSH levels	N/A
	Ding 2017	POA	ICR mice CTX treatment (light, medium, high CTX doses)	hAMSCs hAECs	N/A	PTP: 28d (as compared to hAECs) ↑Follicle counts, AMH and E2 levels (high does CTx), No. of offspring ↓FSH level	No comparison with control or untreated POF mice was shown
	Gan 2017	IUA	SD rats Mechanical injury	hAMSCs	PTP: 7d ↑bFGF, IL-6, VEGF ↓TNF*α*, IL1*β*, TGF*β*, TIMP, COL1A	PTP: 7d ↑Endometrial thickness and no. of glands ↓Fibrotic areas	Long-term implications on fertility cannot be drawn
	Yin 2017	POF	Balb/c mice pZP3 treated Autoimmune injury	hPMSCs	N/A	PTP: 14d ↑No. of mice with regular estrous cycles, E2 and TGF-*β* level No. of primary and secondary follicles ↓FSH and IFN-y level	PTP: 14d No increase in no. of primordial follicles No decrease in no. of atretic follicles
	Ding 2018	NOA	C57BL/6 Aging mice with irregular estrous cycles	hAMSCs	PTP: 28d ↑EGF, HGF	PTP: 28d ↑No. of primordial, primary, ↓secondary, antral follicles per ovary, E2, AMH conc FSH conc	No data on no. of offspring or estrous cycles
	Ling 2019	POI	SD rats-CTX treated	hAMSCs hAMSC-CM	hAMSC-CM ↑Bcl-2, VEGF ↓Bax	PTP: 14, 28, 56d, (both treatments) ↑No. of primordial, primary, and secondary follicles, AMH and E2 level ↓No. of atretic follicles, FSH level, % of rats with irregular estrous cycles, No. of apoptotic GCs	No differentiation into oocytes and hGCs
	Liu 2019	POA	Balb/c mice H_2_O_2_ treated	hAMSCs DES	PTP: 7, 14d ↑VEGF, FSHR, IGF-1, FOXL2, OCT4, GDF9, LIF ↓TNF-*α*, IL-1*β*, SCF	PTP: 14d ↑No. of primordial, primary, and secondary follicles, estrogen levels, and fertility rate ↓Abnormality of estrous cycle, no. of atretic follicles, FSH	No differentiation into oocytes and hGCs
	Feng 2020	POI	SD rats CTX treated	hAMSC in-situ hAMSC tail-vein	PTP: 28d ↑JNK2, P38, Serpin E1	PTP: 28d ↑No. of secondary and mature follicles Serum AMH and E2 level ↓Serum FSH level	PTP: 28d No decrease in % of rats with abnormal estrous cycles
	Kim 2020	POA/NOA	SD rats 52–54 weeks	hAMSCs single injection vs. multiple injections	Multiple injections ↑TGFBR2, ACVR2A, BMPR2, pSMAD1/5	*Single injection, PTP 14d* ↑AMH level Multiple injections, PTP 14, 21d ↑No. of primordial and primary follicles, AMH and E2 levels	Single injection; No increase in follicle count, E2 level Multiple injections; No increase in no. of secondary, preantral or antral follicles
	Cho 2021	POI	SD rats ovariectomized	PD-MSC	PTP: 7, 14, 21, 35d ↑Lhx8, Nanos3, Lin28a, BMP15, EGFR, VEGF, VEGFR, pAKT	PTP: 7, 14, 21, 35d ↑No. of follicles at all stages AMH and E2 levels ↓Atrophy and atresia	No change in no. of arteries, and increase in serum FSH level after 7d
	Liu 2021	AR-DOR	C57BL/6 mice 32 week old	hAMSC	PTP: 7d ↑p-Foxo3a, AMPK, Sod2	PTP: 7d ↑AMH level, no. of secondary and antral follicles, blastocyst formation rate ↓Granulosa and stromal cell apoptosis	PTP: 7d No difference in no. of oocytes, follicle counts, FSHR expression or FSH, E2 levels
Human amniotic epithelial cells cells	Wang 2013	POF/POI	C57BL/6 WT mice Bu/Cy treated	hAECs	PTP: 61d ↑FSHR	PTP: 0, 7, 14, 21, 28, 61d ↑Oocyte production, follicle counts and AMH level	No differentiation into hGCs
	Zhang 2015	POF/POI	C57BL/6 WT mice Bu/Cy treated	hAECs	PTP: 7d ↑Bcl2 ↓TNF-*α*, IL-1*β*, TRADD, Casp-3, Bax	PTP: 28d ↑No. of mature follicles, No. of offspring ↓No. of atretic follicles, inflammation, and follicular atresia	PTP: 28d No increase no. of primordial or primary follicles
	Yao 2016	POI	C57BL/6 WT mice Bu/Cy treated	hAECs hAECs-CM	↑VEGFR1, VEGFR2 ↓VEGFA	PTP: ~ *2-30d* ↑No. of primordial, primary, antral follicles, litter per pregnancy, MVD	*PTP:* ~ *2-30d* No increase in no. of secondary follicles
	Zhang 2017	POF/POI	C57BL/6 WT mice Bu/Cy treated	hAECs hAECs-CM	PTP: 30d ↑MVH, HAS2, BMP15	PTP: 30d ↑AMH mRNA expression, No. of primordial, secondary, and mature follicles	PTP: 30d No difference in no. of primary follicles
	Li 2019	IUA	Balb/c mice Mechanical injury	hAECs	↑ER, VEGF, PCNA, LC3 ↓p62	PTP: 8d ↑Thickness of the endometrium, No. of implanted fetuses, MVD ↓Fibrotic areas	No difference in progesterone receptor expression
	Zhang 2020	POF/POI	SD rats CTX treated	In situ hAECs IV-hAECs	↑IRF7, MX1 in both groups	PTP: 7d ↑No. of primordial, primary, antral follicles, AMH level, No. of rats with normal estrous cycles, No. of fetuses ↓No. of atretic follicles, FSH level	PTP: 14d No increase in no. of antral follicles
	Ouyang 2020	IUA	SD rats Mechanical injury	hAECs uterine hAECs vein	↑bFGF, VEGF, IGF-1, Wnt5a, Snai2 ↓COL1A1,TIMP1,TGFβ, PDGF-C, THBS1, CTGF	PTP: 14d ↑No. of embryos, pregnancy rate, Endometrial thickness, BVD ↓Endometrium fibrotic areas	Long-term implications on fertility cannot be drawn
	Fan 2021	CSD	SD rats Mechanical injury	hAECs	PTP: 30, 60d ↑MMP-8, VEGFA, vWF	PTP: 30, 60d ↑Endometrial thickness, BVD PTP: 60d ↑Total no. fetuses in scarred areas/uterine horn	
Human amniotic fluid stem cellsZ	Lai 2013	POF	C57BL/6 mice Bu/Cy treated	hAFSCs	PTP: 60d ↑AMH, FSHR in cells surrounding oocytes	PTP: 60d ↑Restoration of folliculogenesis AMH, FSHR	No differentiation into hGCs
	Xiao 2014	POF	ICR mice Bu/Cy treated	AFSCs BM-MSCs		PTP: 7-31d ↑No. of primordial, antral, primary, secondary, follicles, and estrous cycles ↓No. of atretic follicles	PTP: 35, 42, 49, 56d No difference in total healthy follicles No differentiation into hGCs
	Xiao 2016	POF	ICR mice Bu/Cy treated	hAFSCs		PTP: 2, 3, 8d ↑No. of total follicles ↓Apoptosis, no. of atretic follicles	
	Huang 2020	OPA	C57BL/6 J mice 14 months with irregular estrous cycles	hAFMSCs	↑FOXL2, CYP19A1, MSH4, STAG3, GDF9, AMH, BMP15, FSHR	PTP: 28d ↑No. of primordial, primary, secondary, and antral follicles, AMH, E2 conc ↓FSH level	

*AMH* Anti-Müllerian hormone; *Bu*/*Cy* busulfan and cyclophosphamide; *BVD* blood vessel density; *CSD* Cesarean scar defect; *DES* diethylstilbestrol; *CTX* chemotherapy; *E*2 Estradiol; *FSH* follicle-stimulating hormone; *hGC* human granulosa cells; *hPMSCs* human placental mesenchymal stem cells; *MVD* microvessel density; *N/A* not available; *NOA*/*POA*, natural or premature ovarian aging; *POF*/*POI*, premature ovarian failure or inefficiency; pZP3, *ZP* glycoprotein 3; *SD* rats, Sprague–Dawley rats

Yin et al. [22] and Gan et al. [23] highlighted the immunomodulatory properties of hAMSCs to improve the regeneration capacity of uterine tissue in IUA mouse models generated by mechanical or autoimmune injury (i.e., by injecting pZP3, zona pellucida glycoprotein 3). The zona pellucida (ZP) antigens on oocytes act as sperm receptors and play a significant role in the process of fertilization. An immune response against ZP antigens interferes with follicle development and leads to follicle depletion (Yin et al., 2018). The studies show that the regulation of immune response by hAMSCs plays a key role in the recovery of damaged ovarian tissue [22, 23]. After hAMSC transplantation, the inhibition of pro-inflammatory cytokines (INF*γ*, TNF*α*), induction of anti-inflammatory molecules (TGF-ß), and regulation of the Treg cell population were found critical in improving the number of glands and reducing fibrotic areas in ovaries [23].

In vivo studies demonstrated that hAMSCs harbor the great ability to colonize uterine tissue and ovarian stroma which helps achieve the utilization rate of hAMSCs thus the desired outcomes. However, various strategies were adopted and compared to improve the homing of stem cells, e.g., repeated vs. single transplantation of hAMSCs [24], direct injection of cells into the ovary vs tail injection [25], or use of polymers for efficient transportation of transplanted cells [13]. Direct vs. tail injection via tail did not result in any significant differences in restoring ovarian function [25]. Contrarily, multiple hAMSC transplantations (3X with 10-day intervals) significantly improved physiological outcomes, i.e., serum levels of ovarian hormones (E2 and AMH) and the number of primordial and primary follicles [24]. However, the long-term implications on fertility cannot be drawn from this study as these outcomes were not found persistent throughout the study time frame. Recently, Haung et al. [13] proposed a polymer-based transportation system PPCNg (polyethylene glycol citrate-co–N-isopropyl acrylamide + gelatin) for hAMSCs transplantation to regenerate endometrial tissue in the Sprague–Dawley rat IUA model. The utilization of PPCNg-based transportation improved the retention of hAMSCs in ovarian tissue leading to the enhance regeneration capacity of endometrial tissue.

In a few comparative studies, the therapeutic potential of hAMSCs in recovering ovarian functions was found superior to other cells such as hAECs [21, 26] or adipose tissue-derived MSCs [27]. The authors found that hAMSCs exhibited a stronger restorative effect than hAECs in mouse models treated with high and medium chemotherapy doses. While in the mouse model generated with a low chemotherapy dose, both hAECs and hAMSCs performed equally, suggesting that hAMSCs might prove superior over hAECs. The hAMSCs transplanted group exhibited a significantly higher number of offspring than the hAEC transplanted group [26]. The distinct effect of hAMSCs seen on restoring ovarian function was attributed to the better biological and molecular characteristics of hAMSCs such as higher expression levels of telomerase, collagen molecular, and stem cell-specific markers. In the subsequent study by the same group [21], the paracrine factors EGF and HGF, secreted by hAMSCs restored the hormonal level and follicle counts better than hAECs. In another comparative study, hAMSCs were found therapeutically more effective than adipose tissue-derived MSCs as evidenced by higher levels of E2 and lower levels of FSH after 2 and 4 weeks of cell transplantation [27].

These inherent characteristics of hAMSCs suggest multiple clinical applications in the field of reproductive biology. However, due to the lack of human studies, clinical uses of hAMSCs are mostly theoretical until now and need further optimization in the preparation and banking procedures.

### Human amniotic epithelial cells (hAECs)

Human amniotic epithelial stem cells (hAECs) have been entertained as another possible source of endometrial regeneration or restoring ovarian function. The hAECs comprise a major portion of the epithelial cell layer of the basement membrane of hAM. The stem cell markers expression profile of hAECs, i.e., CD73, CD90, and CD105 positive, while negative for hematopoietic markers CD34 and CD45) is remarkably similar to MSCs[8]. However, the presence of epithelial-specific cell surface markers such as cytokeratin (CK), E-cadherin, CD49f and EpCAM differentiates hAECs from hAMSCs [28–30]. Higher stability in ex-vivo culture systems and the immunosuppressive nature of hAECs offer great promises in many clinical applications. The possible therapeutic implications for the use of hAECs in the field of reproductive biology are supported by a few recent studies (Table 1).

Wang et al. [31] performed one of the first in-vivo studies to investigate the role of hAECs in restoring ovarian function in chemotherapy-treated mice. Chemotherapy-induced POF results due to loss of pre-granulosa cells of primordial follicles leading to ovulation malfunction. Although hAECs did not present germ cell differentiation markers in in vitro settings, hAECs successfully infiltrated into the ovaries in mouse model, differentiated into granulosa cells, and restored folliculogenesis. The number of secondary follicles per ovary increased significantly in treated mice until 61 days after transplantation. In a few other studies, the paracrine abilities of hAECs to attenuate chemotherapy-induced ovarian tissue damage was reported [32–34]. The angiogenesis, tube formation of hUVECs, and follicle development enhanced remarkably after injecting hAECs into the ovaries of the POI mice model. Mechanistically, hAECs stimulated TGF-*β*/Smad signaling pathway that resulted in a reduction of cell apoptosis thus improving follicle formation [32–34].

Until now, only two studies have assessed the therapeutic efficiency of hAECs for the treatment of IUA (Table 1). Li et al. [35] transplanted hAECs in a murine IUA model established by mechanical injury to the uterus. A significant improvement in clinical parameters such as increased pregnancy outcomes, thicker endometrium, increased endometrial glands and decreased fibrosis was observed in hAECs treated mice. In addition, angiogenesis and stromal cell proliferation marker expression were higher in the hAECs treated group. Similar observations were supported by another study [36]. In this study, hAECs were successfully implanted in a rat model of IAU and resulted in an increased number of embryos and pregnancy rate. In addition, markers for endometrial regeneration (PDGF-C, THBS1, CTGF, Wnt5a, and Snai2), angiogenesis (VEGFA, PCNA) and stromal cells proliferation (ER), and decreased collagen deposition (MMP-8, COL1A1, TIMP-1) were noticed in the treatment group in these studies [35–38] (Table 1).

Infertility or obstetrical infertility complications may occur after cesarean section due to injury to the endometrium and subsequent collagen deposition. In a recent study, the hAECs were used to treat cesarean scar defect (CSD) conditions in a rat uterine scar model [37]. After 30 and 60 days of hAEC transplantation, collagen deposition was reduced, blood vessel density (BVD) was improved, and endometrial tissue was recovered to a great extent. The authors also observed that expression of VEGFA and MMP8, associated with angiogenesis and collagen deposition respectively, increased significantly in the treated group [37]. More importantly, BVD improved significantly in the hAECs group which was attributed in part due to higher expression of a highly specific vascular endothelial marker, von Willebrand factor (vWF). Uterine horns with fetus implantation in scarred tissue were remarkably higher in hAECs than control group suggesting that hAEC transplantation can help regenerate endometrium tissue and may prove a viable therapeutic option for treating uterine scar conditions.

### Human amniotic fluid stem cells

Amniotic fluid is enriched with different types of stem cells in all three phases of pregnancy. Stem cells derived from human amniotic fluid (hAFSCs) are characterized by well-established stem cell surface markers [39, 40]. AFSCs can be isolated by collecting the amniotic fluid at birth or via amniocentesis [41]. The hAFSCs retrieved at full-term pregnancy or delivery is of high therapeutic importance; however, AFSCs retrieved from the second trimester also showed promising results [4]. The cells express germ cell markers such as DAZL (Deleted in Azoospermia-Like gene) and exhibit the ability to differentiate into germ cell lineage [42, 43].

While still in an early stage of development, hAFSC-based in vivo studies show promising results to preserve follicle cells and prevent ovarian dysfunction (Table 1). In a study, the process of folliculogenesis was restored successfully in the ovaries of a chemically induced germ cell-ablated mouse model after hAFSCs transplantation. The evidence of improved folliculogenesis was provided by the augmented expression level of AMH [4]. In another study, hAFSCs repaired follicles by preventing ovary follicle atresia in a chemotherapeutically induced POF mouse model. The number of estrous cycles of hAFSCs treated mice recovered significantly as compared to that of untreated mice. However, the authors suggested improved therapeutic effect could be achieved by timely administering stem cells, i.e., prior to or closer to chemotherapy [44, 45].

In a recent study, mesenchymal stem cells from amniotic fluid (hAFMSCs) were explored to study their potential and mechanism to rescue ovarian senescent cells [46]. The amniotic fluid was collected from the women with a gestational age of 15–22 weeks to isolate MSCs. Following 4 weeks after injection of the cells, levels of AMH and E2 recovered significantly. In addition, hAF-MSCs helped resist ovarian aging by resisting DNA damage as suggested by a decrease in expression of DNA damage markers (e.g., PAPR1, H2AX) after transplantation of hAFSCs.

### Challenges and future directions

Given the ease and availability of placental membranes, which are considered a medical waste following delivery, unexpectedly no clinical studies have been published evaluating the role of amniotic-derived stem cells in restoring ovarian functions in humans. Similar to other fields of medicine, the inherent properties of ASCs warrant their success in the setting of endometrial-driven, chemotherapy-induced, or age-related infertility. However, based on studies reported so far, significant challenges still exist before the translation of these cells in clinical studies. Such as addressing the successful differentiation of ASCs in germ cells in culture, defining the route and time of administration of cells to increase effectiveness, use of precise animal models, and addressing long-term fertility benefits. Future studies should continue to elucidate regulatory mechanisms induced by the amniotic stem cells in ovarian recovery for successful manipulation of these cells in treating infertility.

## Conclusion

Amniotic tissue or fluid-derived human amniotic cells (ASCs: hAECs, hAMSCs, hAFSCs) exhibit stem cell properties with low immunogenicity or tumorigenesis making them theoretically superior to other stem cells. Many studies were performed using mouse models of chemotherapy treated premature ovarian failures, age-related ovarian failure, or other related infertility pathological conditions. These in vivo investigations reported the fundamental findings to understand the mechanisms of actions of ASCs in restoring fertility outcomes. These studies suggest that ASC transplantation promotes follicle formation, endometrial regeneration, glandular development, and restores hormone levels (e.g., AMH and E2). These physiological improvements are carried out due to paracrine, anti-inflammatory, and immune regulatory properties of ASCs. Although these studies provide a theoretical foundation for their application in infertility-related health issues, future studies are warranted to confirm these results for their successful translation into clinical applications.

## Data Availability

Not applicable.

## Abbreviations

AM: Human amniotic membrane
AMH: Anti-Müllerian hormone
ASCs: Amniotic stem cells
BVD: Blood vessel density
CSD: Cesarean scar defect
DAZL: Deleted in azoospermia-like gene
E2: Estradiol
EGF: Epidermal growth factor
FSH: Follicle-stimulating hormone
hAECs: Human amniotic epithelial cells
hAFSCs: Human amniotic fluid stem cells
hAF-MSCs: Amniotic fluid mesenchymal stem cells
hAMSCs: Human amniotic mesenchymal stem cells
hPMSCs: Human placental mesenchymal stem cells
HGF: Hepatocyte growth factor
IUA: Intrauterine adhesion
NOA or POA: Natural or premature ovarian aging
Sox-2: Sry-related HMG box gene 2
vWF: Von Willebrand factor
